# Effects of Unrestricted Kinematically Aligned Total Knee Arthroplasty with a Modified Soft-Tissue Respecting Technique on the Deformity of Limb Alignment in Japanese Patients

**DOI:** 10.3390/medicina59111969

**Published:** 2023-11-08

**Authors:** Masahiro Ishikawa, Masaaki Ishikawa, Hideaki Nagashima, Shinya Ishizuka, Kazuhiko Michishita, Yoshinori Soda, Takafumi Hiranaka

**Affiliations:** 1 Department of Orthopedic Surgery, Nagahama Red Cross Hospital, Miyamae Nagahama, Nagahama 526-0053, Shiga, Japan; ngashima@hotmail.co.jp; 2 Department of Otolaryngology, Head and Neck Surgery, Hyogo Prefectural Amagasaki General Medical Center, 2-17-77 Higashinaniwachou, Amagasaki 660-8550, Hyogo, Japan; 3 Department of Orthopedic Surgery, Nagoya University Graduate School of Medicine, 65 Tsurumaicho Shouwaku Nagoya, Nagoya 466-8550, Aichi, Japan; shinyaishiduka1125@yahoo.co.jp; 4 Department of Orthopedic Surgery, Japan Community Healthcare Organization, Yugawara Hospital, Yugawara 259-0396, Kanagawa, Japan; kmichishita56@gmail.com; 5 Department of Joint Reconstruction and Arthroscopy Center, Midorii Orthopedics, 6-35-1 Midorii, Asaminami-ku, Hiroshima City 731-0103, Hiroshima, Japan; ysoda35@ybb.ne.jp; 6 Department of Orthopedic Surgery and Joint Surgery Centre, Takatsuki General Hospital, 1-3-13 Kosobe-Cho, Osaka 569-1192, Osaka, Japan; takafumi.hiranaka@gmail.com

**Keywords:** total knee arthroplasty, unrestricted kinematically aligned TKA, kinematic alignment, soft-tissue respecting technique

## Abstract

*Background and Objectives*: Unrestricted kinematic alignment total knee arthroplasty (KA-TKA) with a soft-tissue respecting technique (STRT) is a soft-tissue-dependent tibial resection entailing the restoration of the original soft-tissue tension using ligamentotaxis after resurfacing the femur, based on the concept of restoring the native or pre-osteoarthritis alignment in each patient. However, there is no consensus on the indications of unrestricted KA-TKA with the STRT. We modified the STRT, followed by an investigation of the effects of surgery on the postoperative hip–knee–ankle angle (HKAA). *Materials and Methods*: We retrospectively analyzed the clinical background data, including the preoperative and postoperative HKAA, of 87 patients who underwent unrestricted KA-TKA with the modified STRT. Univariate and multivariate analyses were performed to determine the factors affecting the postoperative HKAA. A receiver operating characteristic (ROC) curve was plotted to investigate the change in the cut-off values of preoperative HKAA with respect to the safe zone of the postoperative HKAA. We generated two regression models, the linear regression model and generalized additive model (GAM) using machine learning, to predict the postoperative HKAA. *Results*: Univariate and multivariate analyses revealed the preoperative HKAA as the factor most relevant to the postoperative HKAA. ROC analysis revealed that the preoperative HKAA exhibited a high predictive utility, with a cut-off value of −10°, when the safe range of postoperative HKAA was set at ±5°. The GAM was the superior machine learning model, indicating a non-linear association between the preoperative and postoperative HKAA. Patients with preoperative HKAAs ranging from −18° to 4° were more likely to fall within the ±5° safe range of the postoperative HKAA. *Conclusions*: The preoperative HKAA influences the postoperative HKAA in unrestricted KA-TKA with the modified STRT. Machine learning using the GAM may contribute to the selection of patients eligible for the surgical approach.

## 1. Introduction

Based on global consensus, a neutral coronal limb alignment, defined as a hip–knee–ankle angle (HKAA) of 0°, has been the aim of postoperative limb alignment in total knee arthroplasty (TKA) for decades [1,2,3]. This objective is achieved by mechanical alignment-TKA (MA-TKA), irrespective of the preoperative HKAA, making it a “one-size-fits-all” approach. Several studies have reported good long-term implant survivorship after MA-TKA [4,5]. In MA-TKA, implant survivorship is associated with the postoperative HKAA, with a safe range of 0 ± 3° for better survivorship [1,2,3]. Thus, surgeons currently perform MA-TKA with the aim of having the postoperative HKAA fall within the safe zone.

Recent studies have reported dissatisfaction regarding residual symptoms in 20% of patients after MA-TKA [6,7]. This may be attributed to the fact that MA-TKA aims to achieve neutral coronal limb alignment (0° HKAA) in all cases. One study indicated that an HKAA of 0° is rare in healthy populations, and that constitutional varus is prevalent [8]. This led to the establishment of kinematic alignment TKA (KA-TKA), which is conceptually distinct from MA-TKA [9,10]. Fundamentally, KA-TKA entails restoring the native or pre-osteoarthritis alignment in each patient, making it a personalized or individualized approach. Since no systematic review or meta-analysis has established the safe zone for KA-TKA, surgeons perform KA-TKA with the aim of limiting the postoperative HKAA within the MA-TKA-related safe zone. KA-TKA is categorized into two major types, i.e., restricted and unrestricted KA-TKA [11]. Restricted KA-TKA is based on the concept of restricting the bone cutting angle and is ideally performed under navigation guidance, robotic assistance, or patient-specific instrumentation because it requires precise bone resection to restrict the angle [12,13]. Unrestricted KA-TKA aims to reproduce the pre-osteoarthritis alignment without restricting the bone resection angle. Hence, surgeons may hesitate to employ it in cases with an extreme preoperative HKAA because the postoperative HKAA is theoretically dependent on the severity of deformity in the preoperative HKAA. Elucidating the surgical effects of preoperative HKAA on the postoperative HKAA may aid surgeons in case selection for unrestricted KA-TKA. A crucial concept of unrestricted KA-TKA is to resurface the native articular surface of the femur because it plays a central role in knee motion [14,15]. Different methods may be employed for tibial resection, including the calipered technique and soft-tissue respecting technique (STRT). The main difference between the calipered technique and STRT for unrestricted KA-TKA lies in the concept of tibial resection. The calipered technique is based on the true-measured resection technique, wherein the tibial bone resection plane is determined by estimating the original tibial bone morphology after resurfacing the femur [9,14]. Asians often have proximal tibia vara, which affects constitutional varus limb alignment and is associated with medial tibia bone defects [8,16,17]. Therefore, the use of the calipered technique may result in an excessive residual postoperative tibial component and varus limb alignment in these patients [18]. We conducted a computer simulation study and reported that a residual severe varus tibial component (5° varus KA model) could be correlated with increased contact stress on the medial side [19]. Thus, the calipered technique may yield poor long-term implant survivorship in these patients. Moreover, there is ambiguity about its application in the presence of bone defects as this technique requires estimation of the original bone morphology.

The original STRT entails soft-tissue-dependent tibial resection with the restoration of the original soft-tissue tension using ligamentotaxis after resurfacing the femur [20]. The objective is to revert to the pre-osteoarthritis state by reversing the osteoarthritic changes, which are characterized by cartilage loss and osteophyte formation. This technique is useful for patients with bony defects because the tibial resection level and angle are determined by the soft tissue after resurfacing the femur and resecting the osteophytes affecting limb alignment. Theoretically, unrestricted KA-TKA with the original STRT is more apt for patients with bone defects compared to the calipered technique. However, there have been no studies reporting its clinical outcomes, especially the surgical effects on postoperative HKAA.

Moreover, unrestricted KA-TKA with the original STRT is beset by some technical issues since it requires manual determination of the tibial bone resection level only in extension. Thus, during the first attempt, there may be inter- and intra-operator differences in the tibia resection level owing to the learning curve associated with the surgical procedure. These variations may increase the amount of medial tibial resection, which contributes to the severe varus tibial component that is associated with high contact stress on the medial side (exceeding the required amount). Therefore, we devised the modified STRT to overcome these issues. The critical distinction between the original and modified STRT is that the latter entails cutting the tibial bone using the medial pivot point of the tibia as the reference for the level of medial tibial bone resection. In the modified STRT, the medial pivot point is determined based on both extensions, as in the STRT, and flexion by the Oxford tibial extramedullary resection guide. This procedure may facilitate the accurate determination and reproduction of the medial tibial resection level.

The primary aim of this study was to ascertain the factors affecting the postoperative HKAA in patients who underwent unrestricted KA-TKA with the modified STRT. The second aim was to predict the postoperative HKAA based on the preoperative HKAA using receiver operating characteristic (ROC) curve analysis and machine learning based on regression models.

## 2. Materials and Methods

### 2.1. Patient Selection

The Research Ethics Committee of Nagahama Red Cross Hospital approved the study protocol (approval no. 2023-002). We retrospectively reviewed 87 knees of 74 patients who underwent unrestricted KA-TKA with the modified STRT using posterior cruciate ligament retaining implants (Attune system, DePuy Synthes, West Chester, PA, USA; Vanguard ID system, Zimmer Biomet, Warsaw, IN, USA) at our institution between January 2020 and August 2023. The inclusion criteria were patients who had diagnosed osteoarthritis of the knee. The exclusion criteria were patients who experienced knee collateral ligament injury, history of femoral or tibial osteotomy, and history of trauma around the knee.

### 2.2. Assessments

The following clinical data were collected as explanatory variables for the statistical analysis: (1) age, (2) sex, (3) body mass index (BMI), and (4) preoperative HKAA. Weight-bearing full-length anteroposterior radiographs were obtained at our institution both preoperatively and postoperatively. Radiographic assessment included the mechanical HKAA, lateral distal femoral angle (LDFA), and medial proximal tibial angle (MPTA).

### 2.3. Surgical Procedures

A single surgeon (first author, MI) was the main operator who performed the modified STRT. All decisions about surgical procedures were due to MI. All knee joints were exposed through a small skin incision using the modified subvastus approach.

#### 2.3.1. Distal and Posterior Femoral Resection

The concept of resurfacing each patient’s femur was based on the kinematic alignment principle: the femoral component was aligned in the usual manner, akin to previous studies [9,14]. Briefly, after compensating for cartilage wear, the distal and posterior femur were cut at a thickness equivalent to the femoral component. To restore the original articular surface of the femur, the femur is resurfaced, akin to the conventional callipered technique.

#### 2.3.2. Proximal Tibia Resection

- Original STRT

**Figure 1:**
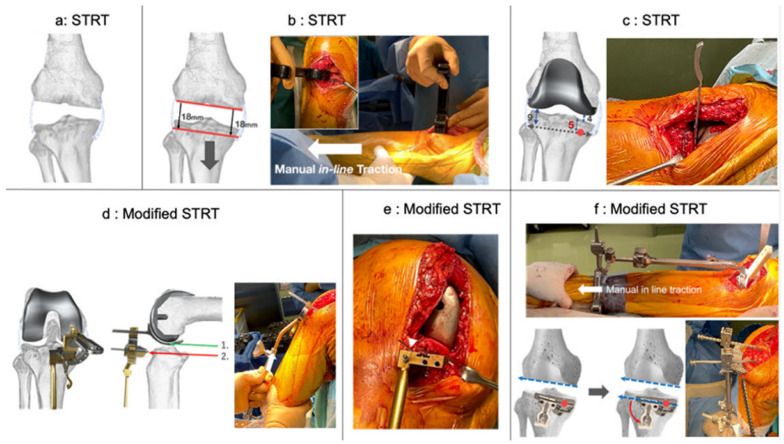
Surgical steps for original STRT. (**a**–**c**): Femoral distal and posterior resection cut at the same thickness as the component following compensation for cartilage wear (**a**). The leg is manually pulled in the distal direction after removing the osteophytes (manual in-line traction technique) in extension. This alignment is defined as the pre-osteoarthritis limb alignment ((**b**) **left**). During traction, the tibial resection level and angle are determined to be parallel to the distal femur in extension ((**b**) **right**). After fitting the femoral trial component, a curved gap gauge is inserted to measure the mediolateral space between the trial component and the tibial bone in extension. The proximal tibial resection level and angle are determined by reproducing the distance (**c**). In addition to the procedure for the original STRT, the resection level of the medial tibia is set at the medial pivot point in extension (red circle). (**d**–**f**): The modified STRT is performed using the medial pivot point of tibia in flexion as reference. A spoon linked to the Oxford extramedullary tibial saw guide connected with G-clamp is inserted under the femoral trial component in 100° flexion. The Oxford guide is grasped and brought up to restore native tension of the medial collateral ligament in flexion (white arrow) (**d**). A 1.2 mm K-wire is inserted to confirm the level of medial tibial resection, i.e., the medial pivot point in flexion ((**e**) the white arrow indicates the medial pivot point). The Oxford guide is replaced with a conventional tibial extramedullary cutting guide. After this step, the resection level of the lateral tibia is determined to be parallel to the distal femur in extension with reference to a fixed medial pivot point [(**f**); the red circle indicates the medial pivot point of the tibia (**bottom left**)]. In flexion, the resection level of the lateral tibia is confirmed again as the thickness of the tibial component from the lateral tibial cartilage surface (**bottom right**). Abbreviations: STRT, soft-tissue respecting technique.

After femoral resurfacing, the femoral and tibial osteophytes were carefully removed (Figure 1a). Thereafter, the lower leg was extended by pulling in the distal direction using the manual in-line traction technique [20,21]. This position was denoted as the pre-osteoarthritis alignment of each limb (Figure 1b). During traction, the first check of the tibial cutting level and angle was performed in extension, which determined if the tibial cutting level and angle were parallel to the distal femur. After fitting the femoral trial component, a curved gap gauge was inserted to measure the medial and lateral space between the trial component and the tibial bone in extension (Figure 1c). Subsequently, the proximal tibial resection level and angle were determined by reproducing the distance, i.e., the second check of the cutting level and angle. Typically, in a varus knee, the thickness of medial and lateral tibial resection is 5 and 9 mm, respectively, when the medial and lateral distance of the trial component–tibial bone is 4 and 0 mm, respectively, and the thickness of the tibial component is 9 mm.

- Modified STRT using the medial pivot point as reference

First, the medial pivot point in extension was designated to be the resection level of the medial tibia per the conventional STRT (Figure 1c). Thereafter, we confirmed the level of medial tibial resection in flexion. After fitting the femoral trial component, a spoon linked to the Oxford extramedullary tibial saw guide was inserted under the trial component in 100° flexion using a G-clamp (Figure 1d left). Generally, we used a 4 G-clamp, which indicates a distance that is 9.5 mm distal to the lowest point of posterior condyle of the femoral component under the native medial collateral ligament (MCL) tension (green: lowest point of posterior condyle of the femoral component, red: 9.5 mm distal level). The Oxford guide was grasped and brought up to restore the native tension of the MCL in flexion (Figure 1d right). In this condition, a 1.2 mm K-wire was inserted to confirm the level of medial tibial resection as the medial pivot point in flexion (Figure 1e). If the medial pivot point differed in extension and flexion, we deemed the intermediate height as the medial tibia resection level. Thereafter, we exchanged the Oxford guide for a conventional tibial extramedullary cutting guide (Figure 1f). After this step, the resection level of the lateral tibia was determined to be parallel to the distal femur in extension with a fixed medial pivot point (Figure 1f, bottom left). The resection level of the lateral tibia was confirmed again as the thickness of the tibial component from the lateral tibial cartilage surface in flexion (Figure 1f, bottom right). The posterior tibial tilt angle was referenced parallel to the AP axis of the base of the medial intercondylar ridge of the tibia (medial slope line), where the cartilage remained in most cases.

### 2.4. Data Analysis

Quantitative variables were presented as the median with the interquartile range or mean with a 95% confidence interval (CI). Age, BMI, and preoperative HKAA were determined to be continuous variables, while sex was deemed a dichotomous variable. To confirm whether the sample size in our data can be sufficient or not statistically, we estimated the sample size required for the study using a paired *t*-test. We calculated the mean values and standard deviations in the changed values of the HKAA at the postoperative time point. A power of 90% and a 2-sided alpha level of 0.05 were set for the analysis. We performed univariate and multivariate analyses to investigate the factors affecting the postoperative HKAA. Age, sex, BMI, and the preoperative HKAA were set as explanatory variables, while the postoperative HKAA was designated as the response variable. Univariate analyses were conducted using a linear regression model (LRM) for the continuous variables, while Fisher’s exact test was employed for the dichotomous variable. Multivariate analysis was also conducted using the LRM.

There is no consensus on the safe range of postoperative HKAA for KA-TKA [22]. Therefore, we assumed several safe ranges and used ROC curve analysis to determine the area under the curve (AUC) and the respective cut-off value of preoperative HKAA within each safe range postoperatively. The optimal cut-off values were determined using the Youden index.

It still remained unclear whether the association between preoperative and postoperative HKAAs can be linear or not. We used two regression models, i.e., the LRM and generalized additive model (GAM), to predict the postoperative HKAA based on preoperative value in unrestricted KA-TKA with the modified STRT. The LRM represents the linear association between the response and explanatory variables, while the GAM represents the non-linear association between them. The issue of using all data for the analysis is the overfitting. To avoid this issue, we used machine learning, and then determined the better model to predict the postoperative HKAA. All data were divided into training (80% of observations) and test datasets (20% of observations). Using the training dataset, we introduced the explanatory variables most relevant to the postoperative HKAA into the LRM and GAM and calculated Akaike’s information criterion (AIC). We calculated the root mean squared error (RMSE) using the test dataset. The model showing lower AIC and RMSE was considered to be the better model and was used as the final model to predict the postoperative HKAA.

The statistical significance level was set at 5%. All statistical analyses were conducted using commercially available software (version 3.6.1; R Foundation for Statistical Computing, Vienna, Austria).

## 3. Results

### 3.1. Patients’ Background Characteristics

The final analysis included 87 knees from 74 patients. The median (IQR) age, BMI, and preoperative HKAA were 77 (72–81) years, 25.8 (22.9–27.8) kg/m^2^, and −7 (−11 to −3)°, respectively. The study sample included 17 men (approximately 20%). There were no cases of tibial plate subsidence and loosening for a maximum of 34 months following surgery.

### 3.2. Radiographic Evaluation and Estimation of Appropriate Sample Size

In all patients who underwent unrestricted KA-TKA with the modified STRT (*n* = 87), the preoperative values of the HKAA, LDFA, and MPTA were −6.8° ± 7.3°, 88.0° ± 2.1°, and 84.8° ± 3.0°, respectively. The postoperative HKAA, LDFA, and MPTA values were −2.2° ± 3.3°, 88.1° ± 1.6°, and 86.5° ± 2.4°, respectively.

The mean values and standard deviations in the changed values of the HKAA at the postoperative time point were 4.6° and 6.1°, respectively. At least 21 patients in total were required to reach statistical significance, indicating that our sample size is sufficient for the statistical analyses. 

### 3.3. Factors Affecting the Postoperative HKAA

We performed univariate and multivariate analyses to identify the factors affecting the postoperative HKAA (Table 1). Univariate analysis revealed that the preoperative HKAA was the only factor significantly affecting the postoperative HKAA. The results of the multivariate analysis were the same as that of the univariate analysis. Based on the results, we used the preoperative HKAA as the explanatory variable in the subsequent analyses.

### 3.4. Cut-Off Values of Preoperative HKAA within the Safe Range

We calculated the cut-off values of the preoperative HKAA that fell within several safe range values of the postoperative HKAA using the ROC curve analysis (Table 2). The cut-off values varied depending on the safe range. The predictive ability of the preoperative HKAA (AUC > 0.9) was higher when ±5° or ±6° was set as the safe zone. The cut-off values of the preoperative HKAA were −10° and −14° when the safe zones were ±5° and ±6°, respectively.

### 3.5. Prediction of the Postoperative HKAA Based on the Preoperative HKAA

**Figure 2:**
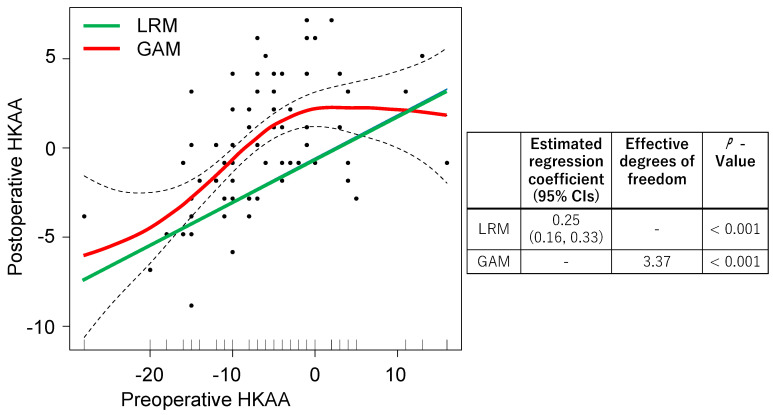
Two regression models showing the association between preoperative and postoperative HKAA. GAM: red line; LRM: green line. The 95% CIs in GAM and LRM are shown as dotted lines and sky-blue ranges, respectively. Abbreviations: HKAA, hip-knee-ankle angle; GAM, generalized additive model; LRM, linear regression model.

We compared all the data using the LRM and GAM to investigate the association between the preoperative and postoperative HKAA (Figure 2). The preoperative HKAA showed a significant regression coefficient in the LRM, while significant effective degrees of freedom were observed in the GAM. For preoperative HKAA values of 0° to 10°, a flat slope was observed in the GAM and a positive slope was seen in the LRM.

Overfitting is an issue if a vast amount of data are used to create the predictive model for the postoperative HKAA. We used machine learning to overcome this issue and compared the AIC and RMSE between the LRM and GAM. The AIC values of the LRM and GAM were 387 and 379, respectively. The RMSEs of the LRM and GAM were 179 and 166, respectively. As the AIC and RMSE values were lower in the GAM compared to the LRM, we concluded that the GAM is a better model to predict the postoperative HKAA.

**Figure 3:**
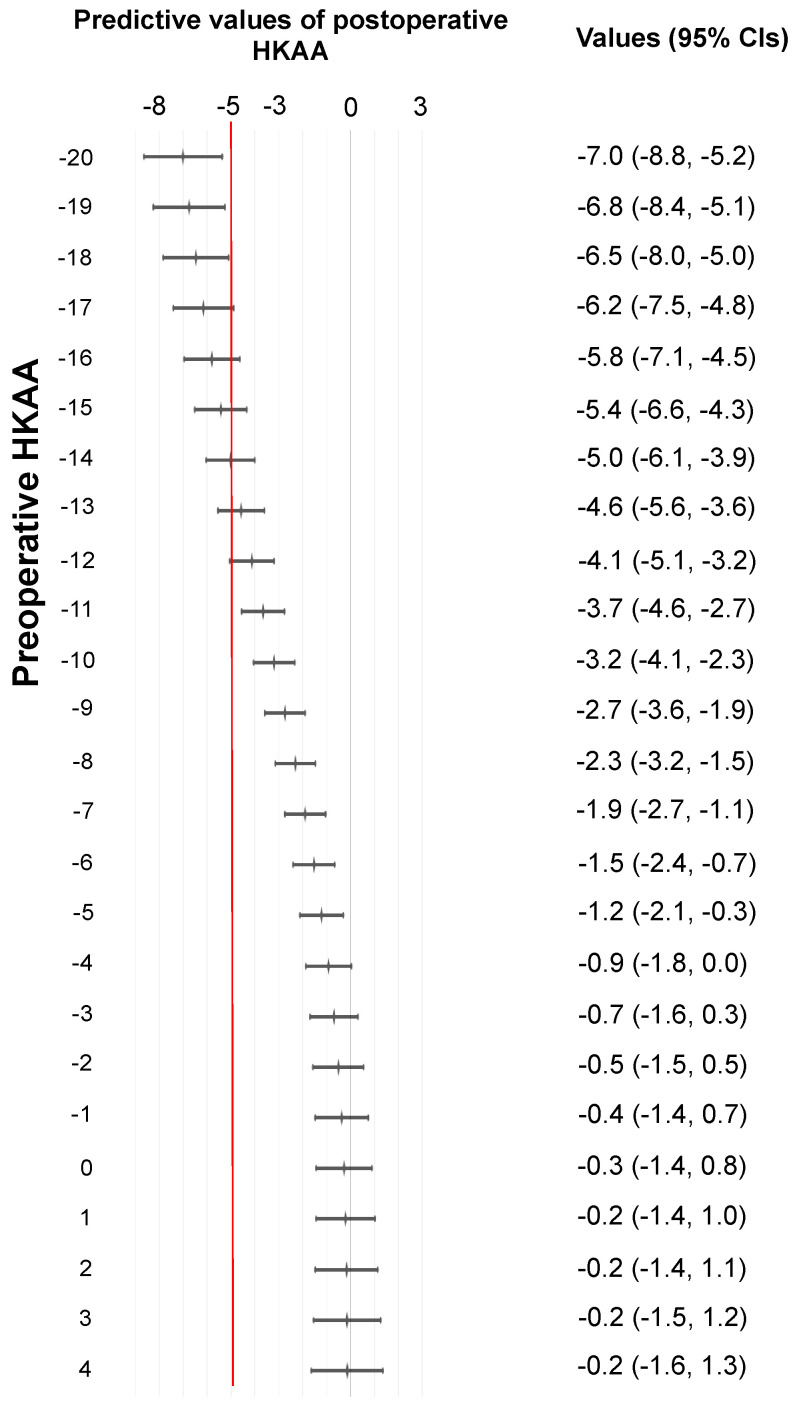
Predictive values of postoperative HKA angle due to preoperative HKAA in the GAM. The red line indicates a postoperative HKAA of −5°. Abbreviations: HKAA, hip-knee-ankle angle; GAM, generalized additive model.

Using the GAM, we predicted the values of postoperative HKAA and plotted the values (Figure 3). The 95% CIs for preoperative HKAAs ≥ −18° and ≤4° ranged from −8° to 1.3° for predicting the postoperative HKAA. When ±5° was set as the safe zone for the postoperative HKAA in unrestricted KA-TKA with the modified STRT, the 95% CIs were located left to the line of −5° of the postoperative HKAA in patients with a preoperative HKAA ≤ −19°. Patients with a preoperative HKAA ≤ −19° are less likely to have postoperative HKAAs that fall within the safe range.

## 4. Discussion

We performed unrestricted KA-TKA with the modified STRT and investigated the surgical effects on the postoperative HKAA. We explored the factors affecting the postoperative HKAA, and the manner in which the postoperative HKAA can be affected by the change in the preoperative HKAA. We found that the preoperative HKAA was the most relevant factor influencing the postoperative HKAA. We used the ROC curve to determine the values of the HKAA that fall within the safe zone. When ±5° and ±6° were designated as the safe ranges of the postoperative HKAA, the respective optimal cut-off values of the preoperative HKAA with a high predictive ability were −10° and −14°. We used the LRM and GAM to investigate the association between the preoperative and postoperative HKAA and found that the latter was the better model, indicating a non-linear association between the explanatory and response variables. The GAM revealed that cases with a preoperative HKAA ≥ −18° to ≤4° can fall within ±5° of the postoperative HKAA.

The difference between the calipered technique and original STRT for unrestricted KA-TKA lies in the concept of tibial resection. The original STRT can be adapted for bony defect cases, which is common in Asian patients, because it is based on the soft tissues’ original length. Therefore, unrestricted KA-TKA with the original STRT is very familiar to surgeons in Japan. However, as the original STRT is performed only in extension based on the manual technique, the surgical outcomes may vary due to the learning curve for beginner or intermediate surgeons. To overcome this issue, we established the modified STRT, which combines the original STRT performed “in extension” with confirmation of the medial tibia resection level “in flexion” using the Oxford tibial extramedullary cutting guide. Please revise it as below. The Oxford guide is customized for the Oxford unicompartmental knee arthroplasty and is usually used to determine the height of the tibial component, which is 9.5 mm distal to the lowest point of the posterior femoral condyle in the flexed position (using a 4G clamp) [23]. Therefore, this cutting guide can indicate the resection level of the tibia automatically. We employed this cutting guide in unrestricted KA-TKA and confirmed the proximal medial tibial cutting level in flexion and performed the modified STRT in extension (defined as the STRT performed with the medial pivot point of the tibia as the reference). The advantage of referencing the proximal medial tibia cutting level in both extension and flexion is that it facilitates the reproduction of the positional relationship between the pre-osteoarthritic femur and tibia on the medial side. Clinical studies have reported that reproducing medial stability is crucial for improving the post-TKA functional outcomes [24]. Theoretically, KA-TKA can reproduce medial stability in the entire range of motion to restore the positional relationship between the pre-osteoarthritic joint line and MCL. Unrestricted KA-TKA has been reported to better reproduce the native mid-flexion rollback and MCL strain compared to MA-TKA [25,26], indicating that restoring the pre-osteoarthritis relationship between the medial side of the femur and tibia may improve knee kinematics. Moreover, unrestricted KA-TKA with the modified STRT requires double-checking by surgeons. Therefore, the modified STRT may contribute to reproducing the pre-osteoarthritic relationship between the medial side of the femur and tibia with lesser intra- and inter-operator variabilities arising from surgical skill because surgeons confirm the medial tibial resection level both in extension and flexion (medial pivot point of the tibia). The indications of unrestricted KA-TKA were hitherto unclear, especially in patients with severe preoperative HKAAs. Our current study provided some interesting findings on the effects of unrestricted KA-TKA with the modified STRT: (1) preoperative HKAA is the most relevant factor affecting the postoperative HKAA, and (2) the application and limits of this procedure derive from the perspective of the preoperative HKAA. A severe preoperative HKAA can theoretically result in a severe postoperative HKAA, consistent with the result of the current study. The GAM contributed to the prediction of the postoperative HKAA in unrestricted KA-TKA with the modified STRT more accurately. At present, there is no specific safe range of the HKAA for unrestricted KA-TKA, and surgeons perform the KA-TKA using the MA-TKA-related safe zone as a reference. A recent systematic review reported that the MA-TKA-related safe range of ±3° is not suitable for modern personalized alignment strategies, such as unrestricted KA-TKA [22]. To overcome this issue, we postulated several safe zones for unrestricted KA-TKA with the modified STRT and calculated the predictive cut-off values of the preoperative HKA angle that would fall within the safe zone of the postoperative HKAA. ROC analyses showed the manner in which the change in the safe zone affected the cut-off values of the preoperative HKAA. A high predictive ability of the preoperative HKAA was observed if the safe zone was set at ±5° and ±6°. Compared with the LRM, the GAM was the better model to predict postoperative HKAA. The main differences in the predicted values between the two models were observed at preoperative HKAA values of −10° to 10°. The GAM showed the steeper slope at preoperative HKAA values of −10° to 0°, followed by a flat slope at ones of 1° to 10° (see Figure 2 and Figure 3). Thus, the effects of unrestricted KA-TKA with the modified STRT on postoperative HKAA can be equable in patients with preoperative HKAA ≥ 1°. Thus, the use of the GAM allowed for a more detailed prediction of postoperative HKAA values from preoperative HKAA values.

It still remains debatable which of the statistical methods between ROC analyses and the GAM using machine learning are suitable for the prediction of postoperative HKAAs, but the limitation of ROC curve analyses can be based on the lack of predicting an actual postoperative HKAA value from each preoperative HKAA one. Considering the lack of consensus on the specific safe zone for unrestricted KA-TKA, the postoperative HKAA predicted by the GAM using machine learning can be more practical for surgeons than the value predicted by the ROC curve because it enabled surgeons to predict the actual postoperative HKAA values. For instance, when surgeons set the safe zone of the postoperative HKAA within ±5°, the target zone can be achieved in patients with preoperative HKAAs ≥ −18° and ≤4° (Figure 3). In other words, the target zone is less likely to be achieved in patients with preoperative HKAAs ≤ −19°. This implies that patients with HKAAs ≤ −19° are poor candidates for the modified STRT, indicating the necessity of using other treatment options.

**Figure 4:**
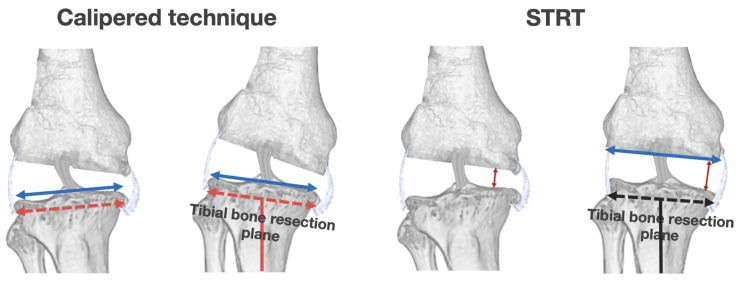
The differences between the calipered technique and the STRT. Calipered technique: The tibial bone resection plane (red dotted line) is determined to be parallel at the base of each tibial spine in an area with intact cartilage (blue line). Hence, tibial resection is made independent of soft tissue. STRT: Medial joint space will be wide following resection of medial osteophytes that affect preoperative HKAA in a varus knee (**left** to **right**). In this condition, the tibial bone resection plane (black dotted line) is determined to be parallel to the distal femur during manual in-line traction. Therefore, STRT is a soft-tissue-dependent tibial resection procedure.

Our prediction GAM may contribute to establishing the surgical indications for unrestricted KA-TKA with the modified STRT with respect to the preoperative HKAA. Patients with preoperative HKAAs ≥ −11° have a high probability of achieving the MA-TKA-related safe zone (±3°). Moreover, it is possible to restore the postoperative HKAA to −5° even in cases with severe preoperative HKAAs ≥ −18° and ≤−12°. This means that the indication of our modified method can be extended even to cases with a severe preoperative HKAA. Surgeons may find substantial results even in cases with severe deformity with our modified method. The concept of tibial resection in the STRT may explain this finding. In the STRT, the tibial resection plane is soft-tissue-dependent, following resection of the medial osteophytes that affect the preoperative HKAA in a varus knee. Recent studies targeting MA-TKA have reported that removing osteophytes is critical for achieving neutral coronal limb alignment [27,28]. Thus, the removal of osteophytes in the STRT may be the key factor underlying the tremendous effects in cases with severe deformity. In contrast, the calipered technique is an independent tibial resection technique; therefore, the tibial resection plane is unaffected by the removal of the osteophytes (Figure 4). The comparison between the calipered technique and STRT may aid an in-depth understanding of the outcomes.

Our study had some limitations. First, this study may have a selection bias because the extent to which the modified STRT would affect the postoperative HKAA was unclear when we started the surgical technique. Hence, patients with slight preoperative limb deformity may have been preferentially enrolled in this study. Second, we investigated the surgical effects of the modified STRT in the present study. Therefore, further studies on KA-TKA with other techniques are needed. Third, a single surgeon was the main operator for the modified approach in all cases. Therefore, it is still unclear whether the modified STRT is less affected by the inter-individual variability in surgical skills than intra-individual variability.

## 5. Conclusions

Our study showed that the preoperative HKAA is the most relevant factor affecting the postoperative HKAA. ROC curve analysis and the machine learning methods revealed the surgical effects of the preoperative HKAA on the postoperative HKAA. Owing to the lack of consensus on a specific safe zone for unrestricted KA-TKA, the results obtained from the GAM may be more practical for surgeons than those of the ROC analysis because the former can predict the actual values of the postoperative HKAA. Our findings may contribute toward the selection of patients who are candidates for unrestricted KA-TKA with the modified STRT.

## Figures and Tables

**Table 1:** Univariate and multivariate analyses to identify the clinical factors affecting the postoperative HKAA.

	Univariate Analysis		Multivariate Analysis	
	Regression Coefficient (95% CIs)	*p*-Value	Regression Coefficient (95% CIs)	*p*-Value
Age	0.05 (−0.04, 0.15)	0.24	0.08 (−0.01, 0.16)	0.07
Sex	0.49 (−1.30, 2.29)	0.59	0.69 (−0.81, 2.19)	0.36
BMI	−0.14 (−0.34, 0.06)	0.17	−0.03 (−0.22, 0.15)	0.70
Preoperative HKAA	0.25 (0.17, 0.33)	<0.001	0.26 (0.17, 0.34)	<0.001

Abbreviations: BMI, body mass index; CIs, confidence intervals; HKAA, hip–knee–ankle angle.

**Table 2:** Comparison of the AUC, sensitivity, specificity, and cut-off values of the preoperative HKAA for different safe ranges of the postoperative HKAA.

Safe Range of the Postoperative HKAA	AUC (95% CIs)	Sensitivity	Specificity	Cut-Off Value of the Preoperative HKAA
±1.0	0.69 (0.57, 0.81)	0.70	0.63	−6.0
±2.0	0.65 (0.53, 0.76)	0.78	0.52	−8.0
±3.0	0.71 (0.59, 0.83)	0.74	0.59	−8.0
±4.0	0.79 (0.66, 0.92)	0.88	0.64	−10.0
±5.0	0.93 (0.87, 0.99)	0.86	0.86	−10.0
±6.0	0.94 (0.87, 1.00)	0.91	0.88	−14.0

Abbreviations: AUC, area under the curve; CIs, confidence intervals; HKAA, hip–knee–ankle angle.

## Data Availability

Data supporting the findings of this study are available from the corresponding author upon request.
